# Targeting Ocular Drug Delivery: An Examination of Local Anatomy and Current Approaches

**DOI:** 10.3390/bioengineering9010041

**Published:** 2022-01-17

**Authors:** Emily Dosmar, Julia Walsh, Michael Doyel, Katlynn Bussett, Adekite Oladipupo, Sabri Amer, Katherine Goebel

**Affiliations:** Department of Biology and Biomedical Engineering, Rose-Hulman Institute of Technology, Terre Haute, IN 47803, USA; walshjr@rose-hulman.edu (J.W.); doyelmj@vcu.edu (M.D.); bussetkn@rose-hulman.edu (K.B.); oladipa@rose-hulman.edu (A.O.); amersm@rose-hulman.edu (S.A.); goebelke@rose-hulman.edu (K.G.)

**Keywords:** drug delivery, anatomy, subconjunctival, intravitreal, subretinal, biomaterials, ocular surface

## Abstract

Ocular drug delivery remains the focus of much modern research. Primary routes of administration include the surface, the intravitreal space, the subretinal space, and the subconjunctival space, each with its own series of unique challenges, limitations, and advantages. Each of these approaches requires careful consideration of the local anatomy, physical barriers, and key cells as well as the interface between the anatomy and the drug or drug system being delivered. While least invasive, the topical route poses a challenge with the many physical barriers that prevent drug penetration into the eye; while injection into the intravitreal, subretinal, and subconjunctival spaces are direct and targeted but limited due to the many internal clearance mechanisms and potential for damage to the eye. Polymeric-based, sustained-release drug delivery systems have been identified as a potential solution to many of these challenges; however, the design and successful implementation of a sustained-release system that is well-tolerated, bioactive, biocompatible, and degradable remains, in many cases, only in the early stages. The drugs and biomaterials in question also require special attention as small chemical changes could result in vastly different outcomes. This paper explores the anatomy and key cells of these four primary drug delivery routes as well as the interface between drug and drug delivery systems and the anatomy, reviewing the recent developments and current state of research in each area. Finally, this paper also examines the frequently used drugs and biomaterials found in ocular drug delivery and summarizes the primary interactions observed.

## 1. Introduction

**Figure 1:**
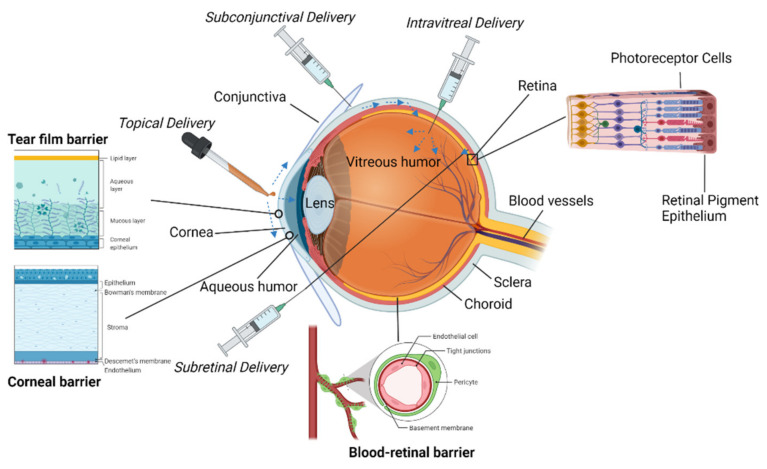
The ocular anatomy, key barriers, and four primary routes of delivery, including topical, intravitreal, subretinal, and subconjunctival. Figure created with BioRender.com (29 December 2021).

Drug delivery to the internal ocular structures remains an important and relevant topic owing to the unique immune privileges of the eye which limit the success of systemic injections. The need to prevent and/or treat several common conditions, including age-related macular degeneration, endophthalmitis, retinal degeneration, diabetic retinopathy, and cataracts, has motivated the development of pharmaceuticals that require entry into the eye and extended residency to be most effective. The ocular surface is a surprisingly diverse portion of the eye, consisting of multiple tissues and glands. From a diagnostic standpoint, ocular surface disease may appear on the inside of the eyelid, on the cornea, on the conjunctiva, and in any of the associated glands. The ocular surface also offers an attractive option for drug delivery due to its relative ease of application and minimally invasive approach. However, the many physical barriers, blood flow, and defensive mechanisms present in the eye limit the ability of traditional eye drops containing drugs to penetrate the internal structures. More invasive alternatives to topical application such as injections and implantable systems through the vitreous, subretinal space, and subconjunctival space have been developed to overcome these challenges, each with its own set of relative advantages and limitations. This paper explores the anatomy and key cells, the biological interface, and the numerous current approaches to delivering ocular pharmaceuticals via the surface, subconjunctival, intravitreal, and subretinal spaces. The various approaches to deliver drugs to each of the aforementioned locations are evaluated and discussed. Figure 1 illustrates some of the key ocular anatomy, barriers, and ports of entry into the eye, including the four delivery routes detailed in this review.

When delivering treatment to the eye, the materials involved must be carefully selected and evaluated to optimize interactions for the desired outcome and minimize potential complications. Where some materials are appropriate for long-term implantation to achieve drug delivery for an extended period, others are better suited for a rapid release, one-time delivery. The drug, treatment, or cells being delivered must also be considered in terms of target location, dosing requirements (volume, frequency, required residency time, etc.), and size. This paper discusses primary material interactions with the ocular space and the unique pharmacokinetics of select anti-VEGF drugs relevant to successful ocular drug delivery.

## 2. Surface

### 2.1. Anatomy and Key Cells 

The surface of the eye has three primary components: the cornea, the conjunctiva, and a protective tear film [1]. Despite their seeming simplicity, this portion of the eye not only is extremely important for vision, but also plays a role in the body’s innate immune system.

#### 2.1.1. The Cornea

The cornea is a highly specialized tissue that serves as a mechanical barrier to prevent microorganisms from entering the eye [2]. Along with the tear film, it is also responsible for the refraction of light as it enters the eye. The cornea is covered in a nonkeratinized stratified squamous epithelium that consists of 5–7 layers of cells [2]. These cells also are connected by tight junctions to keep liquid, toxins, or microbes from entering the intracellular spaces [3]. The cells of the epithelium lie on a basement membrane made primarily of type IV collagen and laminin [3]. Following the epithelium is the Bowman layer or Bowman membrane which is an acellular condensate. This layer will not regenerate if damaged; however, it has been known to scar [3].

Next is the corneal stroma, which provides the structural support for the cornea and most of its thickness. The stroma relies on the precise organization of both the stromal fibers and extracellular matrix for transparency [3]. Keratocytes, generally located in the anterior of the stroma, are the primary cell type and comprise the extracellular matrix found there [3]. A unique characteristic of these cells is that they contain corneal “crystallins”, which are made up of soluble proteins and play a role in reducing the backscatter of light due to the keratocytes [3]. This backscatter reduction is an important part maintaining cornea transparency [3]. The next layer is the Descemet membrane, which is continuously secreted by endothelial cells [3]. The parts of the membrane produced after birth are unbanded and amorphous in structure, while the portions formed *in utero* have a distinctive banding pattern [3].

The final layer is the corneal endothelium which maintains the deturgescence, or dehydrated, state of the cornea, which is necessary for clear vision. The endothelial cells here are initially hexagonal; however, as the cell number drops due to age, trauma, or inflammation, the remaining endothelial cells can stretch to cover gaps that emerge. This stretching causes the cells to grow and lose their hexagonal shape [3]. This is believed to be due to the cornea’s need to maintain a stable metabolic state. As mentioned already, the endothelium is responsible for maintaining the state of the cornea, which includes controlling metabolic inputs and outputs.When cells die, the remaining cells must assume their duties, causing them to take over the functions of the degenerated cells [3]. It should also be noted that corneal metabolism depends heavily on a critical oxygen level and exposure to low oxygen levels may cause permanent morphological changes to the corneal epithelium in addition to damage to the overall corneal physiology [4].

The cornea connects to the conjunctiva at the corneoscleral junction or limbus [2]. The limbus works with the conjunctiva to support the cornea and ensures the conjunctiva does not grow into the cornea. The epithelium of the limbus is continuous with the epithelium of the cornea, making its boundaries difficult to define [2]. The limbus is composed of a nonkeratinized stratified limbal epithelium; however, unlike the conjunctival epithelium, it lacks goblet cells [2].

The limbal epithelium has several layers and contains mature epithelial dendritic cells, immature epithelial dendritic cells, T lymphocytes, and pigmented melanocytes. The basal layer of the limbus contains limbal epithelial stem cells (LESCs), which produce the corneal epithelium [3]. LESCs are located in what is called the limbal niche and are capable of two types of division [2]. During symmetric division, LESCs produce either two new stem cells or two new daughter cells; during asymmetric division, they produce a stem cell and an early transient amplifying cell (eTAC) which can then divide and give rise to the transient amplifying cells (TACs). TACs can migrate and terminally differentiate into corneal epithelial cells [2]. The limbal niche is both vascularized and innervated, unlike the cornea.

#### 2.1.2. The Conjunctiva

The conjunctiva is located on the surface of the eye and the posterior of the eyelids. It is composed of several parts that together with the surface of the cornea are referred to as the conjunctival sac [5]. The bulbar conjunctiva covers the white portion of the eye that is visible or exposed. The palpebral conjunctiva is located on the posterior of the eyelids [5]. The conjunctival fornix (or forniceal conjunctiva) connects the bulbar and palpebral conjunctiva. As a tissue, the conjunctiva is primarily responsible for lubricating the eye [5]; however, it also serves to protect the soft tissues located in the eye, supply immune tissue, and allow the eye to move [6]. The epithelial cells of the conjunctiva contain microvilli which are an important part of allowing the tear film to adhere to the surface of the eye [6]. The conjunctiva also contains a part of the mucosa-associated lymphoid tissue (MALT) called the conjunctiva-associated lymphoid tissue (CALT). This system has all the components needed for a complete immune response [6]. This system can help a tissue introduce tolerance to some antigens, as well as detect antigens, and induce a direct immune response. In addition, the conjunctiva secretes IgA as well as some other components of the secretory immune system [7].

On the cellular level, the bulbar conjunctiva is mostly nonkeratinizing squamous epithelium. Mixed within this epithelium are goblet cells, specialized endothelial cells [8]. Goblet cells produce mucins, highly glycosylated glycoproteins, that allow them to form a mucosal layer atop a tissue [9]. Ocular goblet cells secrete a mucin called MUC5AC, which is a known marker for their identification [9]. This secretion is part of the body’s innate immune response and the failure to produce it may lead to an increased risk of infection [8]. Goblet cells are also noted to secrete RELM-β, which is bactericidal, and zymogen granule protein 16 (ZG16), which aggregates bacteria and stops them from adhering to the body’s epithelium [8].

Recent work has indicated that goblet cells are able to pass antigens to dendritic cells through what is called goblet cell-associated antigen passages (GAPs), which can lead to an inflammatory response [10]. This has led to the conclusion that goblet cells play a significant role in immune tolerance of the ocular surface, and immune tolerance can be lost when goblet cells are absent [10]. Other cells found in the bulbar conjunctiva are Langerhans cells, melanocytes, and lymphocytes [6]. The bulbar portion of the conjunctiva relies on tight junctions, gap junctions, and desmosomes for selective permeability. This is all built on a basement membrane of type IV collagen which rests on the substantia propria, a loose connective tissue with high vascularization [6]. This is in turn loosely connected to the underlying Tenon’s capsule [6].

The conjunctival fornix is continuous with the skin while also connecting the bulbar and palpebral conjunctiva. It contains nonkeratinized stratified squamous epithelium [6] in three layers: cylindrical, polyhedral, and cuboidal [6]. Goblet cells, melanocytes, and dendritic cells are also interspersed [6]. The substantia propria of this portion is thicker since it contains two parts: a superficial lymphoid layer, which contains connective tissue with lymphocytes, mast cells, plasma cells, and neutrophils, and a deeper fibrous layer that contains nerves, vessels, and the glands of Krause.

The palpebral conjunctiva lines the inner surfaces of the eyelids, extending from the mucocutaneous junction of the eyelid to the fornices [6]. The palpebral conjunctiva is nonkeratinized stratified squamous epithelium and contains cuboidal epithelial cells and columnar epithelial cells. It also contains Langerhans and goblet cells [6]. The palpebral conjunctiva contains regions: the marginal, tarsal, and orbital conjunctiva. The marginal conjunctiva contains the glands of Walfring and is where the transition from nonkeratinized stratified epithelium of the eyelid to cuboidal epithelium of the tarsal conjunctiva occurs [6]. The tarsal conjunctiva also contains infolds containing goblet cells which are called the pseudo glands of Henle [6]. The orbital conjunctiva extends all the way to the fornix and folds when the eye opens.

#### 2.1.3. The Tear Film

The tear film of the eye, composed of water, mineral salts, antibodies, and lysozymes, is essential to ocular function and health [11]. The tear film plays a role in clear vision, maintains epithelial cell health, and is part of the body’s innate immune response [11]. It creates a smooth surface for refraction, supplying approximately two-thirds of the refraction of the eye [11], and is responsible for eye lubrication, a very necessary component of comfort. A healthy tear film is approximately 6 μm thick and protects against potential irritants as well as flushing the eye when needed [11]. Foreign bodies and other irritants will increase the production of tears as part of the body’s self-defense. Tear production is a result of a reflex loop that is driven by the nerves of the ocular surface, central nervous system, and glands of the ocular surface, sometimes collectively referred to as the lacrimal functional unit [11].

There is a noteworthy chemical distinction between tears caused as part of an emotional response and those stimulated by an irritant. Protein-based hormones, prolactin, adrenocorticotropic hormone, and leucine encephalin, all compounds produced under stressful conditions, are more predominantly present in emotional tears as a mechanism to expel them from the body. The three major components of tears are made up in three distinct anatomical layers: oil, water, and mucus produced by the meibomian glands which line the edge of the eye, the lacrimal gland underneath the outer orbital rim bone, and the goblet cells in the conjunctiva, respectively [12]. Tear film is spread evenly across the surface of the eye with every blink, and the motion then forces the tears into the puncta (drains) located in the corners of the upper and lower eyelids [12]. Tear film travels from the puncta into the upper and lower canaliculi which empty into the lacrimal sac; from there, it is drained into the nasolacrimal duct that connects to the nasal passage [12].

The tear film contains several different antimicrobials such as peroxidase, lactoferrin, lysozyme, and immunoglobulin A [13]. The tear film also contains glucose, electrolytes, and growth factors for supporting the cornea, which is avascular. Overall, the composition of the tear film would be a dilute protein solution similar to serum, but with different concentrations of its various parts [11]. There are two sections to the tear film: a lipid top layer that prevents evaporation and a mucin gel called the glycocalyx gel beneath that executes the many functions of the tear film. The lipid portion is secreted by the meibomian glands and has a low surface tension to allow the tear film to spread uniformly, with polar lipids preferring to locate themselves against the aqueous layer and nonpolar lipids moving toward the lipid–air interface [11]. As mentioned earlier, the aqueous layer is similar in composition to serum, especially for electrolyte concentration. The main and accessory lacrimal glands, specifically the glands of Krause and Wolfring, produce the aqueous component constantly [11]. The aqueous component of the tear film starts with a very low concentration of mucins near the lipid layer and sees an increase in concentration as it approaches the corneal epithelium. The gel is mostly hydrophobic glycoproteins that help the matrix firmly attach to the corneal epithelium, as well as increasing viscosity and lowering surface tension to help with keeping the hydrophobic ocular surface wet uniformly [6,11]. Transmembrane mucins, which help with anchoring the matrix, are found in the corneal and conjunctival epithelium [11]. The microvilli of the squamous epithelium of the cornea interact with the mucins in the tear film, helping to anchor and stabilize the tear film [11]. There are cell-surface-associated mucins that form the glycocalyx, while secreted mucins are either soluble (closed to the tear film limit layer) or gel-forming (located near the conjunctival apical cells) [6].

The meibomian glands are responsible for producing the oils that keep the aqueous portion of the precorneal tear film from evaporating [5]. They are located in a portion of the eyelid called the tarsus, which is located behind the eyelashes. On average there are 25 meibomian glands in the upper eyelid and 20 in the lower eyelid [5]. During the development, they differentiate from the pilosebaceous unit, same as eyelash follicles, so there are some conditions where this gland can be replaced by an eyelash [5].

The glands of Krause are located in the conjunctival fornix. They are an accessory lacrimal gland, with 42 located in the superior fornix and 6–8 located in the inferior fornix. They, like the main lacrimal gland, produce the aqueous component of the tear film [6]. The glands of Wolfring, also known as the glands of Ciaccio, are located within the palpebral conjunctiva, specifically above or within the tarsus [6]. They are another minor accessory lacrimal gland and also produce tears [6].

Tears act as a vehicle for the delivery and excretion of nutrients and metabolic products of the corneal epithelium and anterior stroma [14]. The quality of vision is also a function of the stability of the tear film which keeps the surface of the eyes smooth and clear and serves as a protective barrier to infectious agents [15]. The viscosity of tears is low (1–10 mPa-s) and is known to exhibit non-Newtonian properties, thus having a dependency on sheer rate [14]. The reported flow rate for normal tear flow is approximately 1.2 µL/minute [16], is driven by a pressure gradient, and is influenced by the rate of evaporation and production [17]. A 2009 study examined the contribution of tangential flow to tear thinning and breakup between blinks and found that this flow is generally too slow and thus evaporation accounts for the bulk of tear thinning [17]. Pressure gradients and gravity also give minor contributions to this event [17]. A viscoelastic component of the lipid layer of tear fluid has been modeled to describe the upward movement of the fluid after a blink [17]. Once the uneven lipid tension driving the movement becomes nearly uniform, the movement terminates [17]. A Reynolds number has not been reported for tear flow, but a relatively low value is suggested by the assumed laminar flow used in models [17].

The eye also must drain the fluid from its surface. The lacrimal drainage system passes liquid from the eye to the nose. Liquid enters the puncta, two drainage points located on the posterior of the eyelid margin, one on the upper and one on the lower lid [5]. These look like small indents when an eyelid is observed in the mirror. These pass the liquid to the canaliculi, tubes that eventually fuse before meeting the lacrimal sac [5]. The lacrimal sac can store some fluid and connects to the nasolacrimal duct which allows fluid to exit the nose by way of the inferior turbinate [5].

### 2.2. Interface

A drug is typically delivered to the eye in the form of a free drug in an aqueous suspension administered as a liquid drop or ointment. Most surface drug delivery is noninvasive, is placed directly onto the surface, and diffuses directly into the eye. The main issue with these carrier systems is that ocular tissue is highly sensitive, and an incorrectly formulated drug or delivery mechanism will lead to ocular irritation, inflammation, and vision interference [18]. The ocular surface has several natural barriers to drug absorption. Drainage abilities of the ocular surface clear many drugs before they can be absorbed, absorption by the conjunctiva is nonproductive, and the cornea is lipophilic which complicates the delivery of hydrophilic compounds [19].

Precorneal fluid drainage is a main cause of low ocular drug absorption [20]. After administration, approximately 80–95% of the initial dose volume is drained into the nasolacrimal duct which is meant to help maintain the precorneal fluid volume to about 7–10 µL [21]. Along with excess fluid presence, addition of a fluid with pH varying from 7.4 (the pH of tear fluid) will result in excessive tear secretion and loss of drug [20]. Tears will also dilute any hypertonic solutions they encounter, requiring the treatment solution to be isotonic with tears [20].

The diffusion of a drug into the eye is controlled by the epithelium of the cornea. Due to the lipoidal nature of the epithelium, the treatment solution must exhibit intermediate solubility in the lipid layer to be effectively absorbed [22]. The lamellar stroma is predominately aqueous, which requires the treatment solution to exhibit intermediate solubility in the aqueous layer and lipid layers for effective absorption [23]. The presence of leaky tight junctions can allow for the passage of macromolecules across the corneal epithelium and is mediated by local osmotic gradients as well as the sodium pump [24]. However, even if the applied drug can diffuse through the corneal epithelium, the treatment often fails to reach the retina and vitreous humor with sufficient concentration [23,25,26,27].

The conjunctiva and sclera are considered minor pathways for drug delivery compared to the corneal route. Transport of hydrophilic solutes across the conjunctiva is limited due to tight junctions between epithelial cells [28]. The sclera, consisting of mostly collagen, is more permeable than the cornea, but less so than the conjunctiva [23]. Ocular drugs can be absorbed via the conjunctiva and delivered to the eye via the sclera, but this route is considered nonproductive due to the drainage loss through blood vessels in the conjunctiva [23].

#### 2.2.1. Topical Liquids and Solutions

Topical liquids are popular due to their relatively noninvasive mode of delivery [18]. Their most common form is eyedrops [18]. Through topical solutions, a drug is administered into the precorneal pocket; however, typically only 0–20% of the administered drug is retained, and the rest is lost to blinking [18,25,29,30,31,32,33]. In addition, the potency, bioavailability, and clearance of the drug at the target ocular tissue are all factors that affect parameters such as required drug loading, release rate, and ocular retention times of drug delivery systems [34]. Additionally, the material properties and size constraints of the eye limit drug-loading capacity [34].

To treat conditions that affect the cornea and conjunctiva or tissues surrounding the anterior chamber (e.g., anterior segment diseases, inflammation, minor infections), it is typically sufficient to apply the drug directly to the ocular surface via eye drops, where the drug will mix with the lacrimal fluid. However, to be effective, the drug must remain in the tear film or become absorbed by the cornea or conjunctiva [34]. If intraocular tissues such as the trabecular meshwork, iris, or ciliary body are the target of the drug, then it is necessary for the drug to permeate through the cornea and conjunctiva. Topical drug application is typically not effective for administering a drug to intraocular tissues, as effective concentrations of the drug do not reach the posterior segment. In most cases, topically applied drugs can permeate across the cornea but travel no deeper than the aqueous humor. Tight junctions in the corneal epithelium majorly restrict drug absorption [35], but drugs diffuse freely through the corneal stroma and corneal endothelium [36]. Once a drug reaches the aqueous humor, it can diffuse easily to the intraocular tissues. However, the distribution of drugs further into the vitreous and retina is limited by the physical lenticular barrier, aqueous humor turnover, and blood flow in the iris and ciliary body [37].

Aqueous humor turnover and the blood flow in the iris and ciliary body are sufficient to eliminate small-molecule drugs, and aqueous humor turnover can clear large-molecule drugs [38,39]. Hydrophilic small-molecule drugs can diffuse across the conjunctiva and sclera from the ocular surface to the iris and ciliary body without entering the aqueous humor. It is possible that large molecule drugs can also enter the iris and ciliary body via this route, as the openings in the conjunctival epithelium are larger than those in the cornea [35,40,41].

Depending on molecular weight, hydrophobicity, size, etc., drugs can passively diffuse across the cornea. Various additives can be added to topically applied drugs to improve their contact time, permeation, and ocular bioavailability. These additives include viscosity enhancers, permeation enhancers, and cyclodextrins [18]. Viscosity enhancers improve precorneal residence time and bioavailability [18], which can help reduce drug loss due to blinking. Permeation enhancers slightly compromise corneal integrity to improve corneal uptake and drug bioavailability, but some studies have shown that they can cause local toxicity [18]. Cyclodextrins can carry hydrophobic drugs in aqueous environments, which aids in delivering hydrophobic drugs to highly lipophilic biological membranes [18]. The lipophilic membranes have a low affinity for the cyclodextrins themselves but a higher affinity for the hydrophobic drugs, which causes the cyclodextrins to remain in the aqueous solution when the drug is absorbed by the membrane [18].

#### 2.2.2. Emulsions and Microemulsions

Emulsions are colloidal systems with improved stability and drug bioavailability as compared to topical medications [18]. There are two types of emulsion systems: oil in water (o/w) and water in oil (w/o) [18,42,43,44]. For optical drugs, o/w systems are preferred, as they cause less irritation and are better tolerated than w/o systems [18]. Emulsions are known to increase the bioavailability, permeation, and residence time of the drug they are delivering [18,44].

Microemulsions are such systems between 5 and 200 nm and show significant thermodynamic stability, low surface tension, and enhanced drug retention time leading to greater absorption [44]. Microemulsions are a particularly attractive option for ocular drug delivery due to their effectiveness at delivering poorly water-soluble drugs [44,45,46,47,48,49,50,51] and their optical transparency [52]. They have been effective in delivering drugs targeted to treat glaucoma, uveitis, keratitis, and bacterial and fungal infections of the eye [44]. Unfortunately, the significant downside associated with the use of microemulsions is the large quantity of surfactant required to form stable microemulsions. A high concentration of surfactant on the surface of the eye could cause ocular toxicity. Depending on the particulars of a case, the use of a nonspontaneous preparation process in conjunction with coarse emulsions may be justified to reduce the risk of ocular toxicity. This issue can also be remedied by pursuing nonionic surfactants such as sugar ester surfactants and polysorbates such as Tween 60 and Tween 80 [44,53,54,55,56] which reduce both toxicity and ocular irritation [44].

#### 2.2.3. Suspensions and Nanosuspensions

Suspensions are used for the delivery of insoluble pharmaceuticals, generally by dispersing them in an aqueous solvent [18]. Particle size is a substantial indicator of drug residence time and activity; small particles in the precorneal pocket replenish the drug absorbed by ocular tissue, while large particles are more easily retained in the precorneal pocket and slow drug dissolution [18]. Suspensions require a “dissolution or release” of a drug prior to absorption [57]. Release, ocular residence time, and bioavailability of a drug all vary based on the physicochemical properties of the suspension [58]. In a rabbit model, Vooturi et al. investigated budesonide solutions at different viscosities and determined that an increase in viscosity significantly improved the ocular bioavailability to the aqueous humor [57].

Nanosuspensions have similarly been shown to improve the bioavailability of hydrophobic drugs by improving solubility and residence time, with the only drawback being physical stability and the potential for drug sedimentation [59]. Ali et al. demonstrated a 1.8-fold improvement in the bioavailability of hydrocortisone when prepared as a nanosuspension as compared to the commercially available solution [60].

#### 2.2.4. Ointments

Ointments can improve bioavailability and sustained release of ophthalmic drugs [18]. An ointment is a mixture of semisolid and solid hydrocarbons that has a melting point at ocular temperature (34 °C) [18]. Biocompatibility is the primary determinant of what hydrocarbon is selected for use in the ointment [18]. Notably, ointments are a prevalent and effective way of delivering the broad-spectrum antibiotic vancomycin through minimally invasive means [61]. As of 2019, at least three phase 3 clinical trials have been completed for vancomycin ointments that are intended to treat such conditions as bacterial conjunctivitis [61,62] and have been previously used to treat blepharitis, conjunctivitis, and keratitis caused by MRSA and MRSE [63].

#### 2.2.5. Contact Lenses and Hydrogels

Contact lenses adhere to the tear film of the cornea using surface tension and were traditionally made of poly(methyl methacrylate) (PMMA); however, more recent lenses are made of hydrogels [18,64]. Soft contact lenses are now a polymer blend (often of silicone and/or polyhydroxyethyl methacrylate (HEMA)) [64]. There are two types of contact lenses: hard and soft. Hard contacts are rigid but gas-permeable; soft contacts are flexible and made of high-water-content materials and are oxygen-permeable, an important feature for maintaining eye health. Softer lenses tend to fit the shape of the eye better due to their flexibility [64].

Drugs delivered by contact lenses have longer residence times in the tear film and continuous drug delivery, leading to more drug entering the cornea [18,30] and the advantage of greater than 50% bioavailability in comparison to traditional eye drop solutions [29,65,66,67]. When designing contact lens drug delivery systems, there are several factors to consider, including lens transparency, oxygen permeability, glass transition temperature, wettability, and water content [30]. Traditionally drugs have been loaded onto contact lenses by soaking them in solution; however, this is not an efficient means of loading and has only a short-term release [18]. This has led to the creation of particle-laden contact lenses, where drugs are entrapped in vesicles dispersed in the contact lens material [18]. For this method, implantation, nanoparticles, liposomes, microemulsion, and micelles are used [29,65,68,69]. These and other novel polymer methods of delivering drugs have also been conceived, including molecular imprinting and use of vitamin E as barriers [29,65,68,69]. However, since these vehicles are generally not covalently bound to the matrix, they can escape and cause irritation [70]. These new methods rely on drug diffusion from the matrix, the degradation of the matrix, or the polymer responding to external stimuli, such as pH or temperature [30,64]. Drug-eluting contact lenses have the unique concern that their optical or mechanical properties could change as the drug is lost; this has led some manufacturers to leave a clear central zone for the pupil [70].

There are several areas of concern for contact lenses. The surface roughness can affect the ability of bacteria to adhere to them, so contact lenses with nanoparticles embedded may have a greater chance of bacterial adherence. Additionally, contact lenses may lose some of the drug they are loaded with during storage, which is something manufacturers need to be aware of when packaging and designing products. Finally, the contact lenses need to be both thin enough that they stay in the eye and transparent enough not to impede vision, which some modifications to lenses may not allow [70].

In 2009, Ciolino et al. developed a solvent cast poly(lactic-co-glycolic acid) (PLGA) sandwich contact lens capable of releasing fluorescein and ciprofloxacin at a steady rate for a month with a minimal initial burst [25]. This approach has since been used successfully to deliver econazole to inhibit fungal growth in vitro [29,71], latanoprost [29,72], and dexamethasone [29,73]. Currently, there are several clinical trials either recruiting participants or under investigation involving eluting contact lenses of these same drugs (clinicaltrials.gov accessed 13 May 2021). Most recent research efforts regarding drug-eluting contact lenses have looked towards developing bioresponsive and smart materials that deploy drugs based on received biosignals in vivo [74].

## 3. The Intravitreal Space

### 3.1. Anatomy and Key Cells

The intravitreal space comprises the majority of the eye’s volume and is located behind the lens of the eye [75]. The vitreous chamber of the eye is mostly filled with a gel-like solution called the vitreous body [75]. The vitreous body is 98.5–99.7% water containing salt soluble proteins and hyaluronic acid [75]. This is all contained by a gradient mesh of collagen that decreases in density towards the center of the structure [75]. The hyaluronic acid is located strategically within the collagen network to help maintain the spacing between fibrils and acts as a stabilizer [75]. The hyaluronic acid allows the gel to swell in the presence of water [76]. The water-bound hyaluronic acid allows the vitreous to maintain a gel-like consistency [75]. Hyalocytes, the phagocytic cells that comprise the vitreous body [75], are located in a single layer in the cortex of the vitreous and synthesize hyaluronic acid and glycoproteins. Hyalocytes also have binding receptors for IgG and complement components, which play a role in the immune response and in removing cellular debris [77]. Under certain pathologies, these cells can exhibit similar abilities to macrophages [75,78,79,80]. Their function is dictated by their location inside the vitreous [75]. In addition to hyalocytes, the intravitreal space contains a relatively small number of fibroblasts and macrophages. The fibroblasts are located near the front and back of the eye and are believed to produce collagen fibrils [75]. The macrophages are believed to originate from retinal blood vessels and only occasionally appear in the vitreous [75].

There are three density zones within the vitreous body. The vitreous cortex, also known as the hyaloid surface, is the most superficial zone [75]. It is composed of tightly packed collagen fibrils that run both parallel and perpendicular to the retinal surface [75]. This section runs from the side of the inner eye to the retina and contains several transvitreal channels [75]. The first transvitreal channel is the prepapillary hole, which is visible when the vitreous detaches from the retina, followed by the premacular hole, an area of lower density within the vitreous body. Finally, there are prevascular fissures, which exist where the collagen fibers enter the retina to attach to retinal vessels [75]. The next zone is the intermediate zone, which contains fine collagen fibers running anterior to posterior [75]. The fibers run parallel to the most proximal density zone [75]. This region also contains condensations of different collagen fiber densities, called vitreous tracts [75]. The final and deepest zone is Cloquet’s canal, also known as the hyaloid channel or the retrolental tract. This zone is S-shaped and is a leftover of the hyaloid artery system that was in its position during embryonic development [75]. This zone terminates at the area of Martegiani, a space at the optic nerve that extends forward into the vitreous in a funnel shape [75].

The vitreous chamber is predominantly surrounded by basal laminae, to which the vitreous attaches at several points. Its most notable connections are the vitreous base and the hyaloid capsular ligament of Weiger [75]. The vitreous base connects the vitreous to the basement membrane of the nonpigmented epithelium of the ciliary body and the internal limiting membrane of the peripheral retina [75]. For the retina, this is a continuation of the basement membrane of Müller cells [76]. The vitreous base connects the vitreous to the basement membrane and internal limiting membrane via vitreous fibers that are embedded into these membranes. The full base can extend a couple of millimeters into the vitreous [75]. The hyaloid capsular ligament, also referred to as the retrolental ligament, is an annular ligament located between the posterior surface of the lens and the vitreous. The potential space between these two surfaces is sometimes called the retrolental space of Berger. This ligament loses strength with age, particularly after age 35 in humans [75]. The vitreous is also connected to the macula via peripapillary adhesions around the edges of the optic disc. These adhesions also diminish with age [75]. In addition, fine collagen strands connect the vitreous to retinal blood vessels. These collagen strands pass through the internal limiting membrane to connect and surround the larger retinal vessels [75]. It is unclear how the vitreous attaches to the rest of the internal limiting membrane [75].

### 3.2. Interface

The intravitreal space is often used as a delivery site to treat eye diseases of the posterior segment. Techniques for administration of pharmaceuticals to the vitreous or to the posterior of the eye via the intravitreal space vary [81]. This section will explore some of the major approaches.

#### 3.2.1. *Injections*

The first intravitreal injections (IVIs) were developed in 1895 to treat retinal detachment and vitreous hemorrhage [82]. However, since the 1970s, the number of IVIs has exploded, with antibiotics, steroids, gasses, and other compounds being injected once it became clear that IVIs could bypass the blood–retina barrier [82]. IVI is used as a method to achieve maximum drug concentrations in the vitreous and retina [81,83,84,85,86,87]. Under normal circumstances, the injection is accomplished using a 30–32 gauge filter needle, targeting the inferotemporal quadrant to avoid the visual axis [82]. It is believed that injecting more the 100 μL is unsafe, excluding gas-based treatments [82]. The needle is removed after injection, and a cotton-tipped applicator is placed over the injection site to reduce reflux for injections larger than 0.05 mL [82]. The use of antibiotics is a bit varied, with some groups preferring to skip their preoperative application [82]. Antibiotics help prevent complications such as endophthalmitis; however, there is some evidence of cases where antibiotics may not be needed [88,89]. The most common complications for IVIs are ocular pain, subconjunctival hemorrhage, and elevated intraocular pressure (IOP) [82]. They do also carry the risk of more severe conditions, such as subretinal hemorrhage, retinal toxicity, and retinal detachment, though these disorders are rare [82]. The most significant complication is endophthalmitis, with has a risk range of 0.14% to 0.87% per injection and occurs most commonly when antiviral agents are injected and least commonly when gases are injected [82]. This is believed to be due to the increased frequency of injections needed for antivirals as compared to other compounds [90].

The administration of triamcinolone acetonide via IVI is currently a common treatment for a variety of ocular diseases. Although this method is generally accepted for use in appropriate circumstances, concerns have been reported surrounding potential complications to the vitreous. To further investigate, researchers at the Erciyes University Medical Faculty in Kayseri, Turkey, studied the effects of IVIs in 180 patients [91].

A total of 20 IVIs were administered to the 180 subjects (212 eyes), with 48 subjects’ eyes receiving a second injection and 5 subjects receiving a third injection. Subjects were monitored for 4 weeks after injection via follow-up appointments. One of the most common side effects observed across the patient base was a transient increase in IOP, with the mean IOP spiking approximately 3 months post-injection and returning to preoperative levels (approximately 15 mm Hg) 9 months after the injections. IOP was observed to surpass 21 mm Hg in 44 of the tested eyes. In 14 of the tested eyes with diabetic macular edema, an intraocular lens implantation was required after the injections; however, 10 of these subjects showed previous signs of cataract development. The researchers determined that the continued use of triamcinolone acetonide injections to the vitreous is an effective treatment for appropriate ocular diseases; however, consistent monitoring for dangerous increases in IOP or the development of cataracts is a necessary precaution that needs to be taken when using such injections [91].

Similarly, to evaluate the risk of retinal detachment following IVI, Storey et al. evaluated 180,671 IVIs in 12,718 unique patients that received ranibizumab, bevacizumab, or aflibercept for neovascular age-related macular degeneration or retinal vein occlusion. They concluded that there was no association between the risk of retinal detachment following injection and diagnosis (*p* = 0.54), physician experience (*p* = 0.23), injection site (*p* = 0.41), caliper use (*p* = 0.75), or 31- versus 30-gauge needle use (*p* = 0.18). However, the macular status of the patient at the time of the retinal detachment did have a significant impact on the ultimate visual outcome. Ultimately, the rate of retinal detachment following a single IVI was 1 in 7500 [92].

#### 3.2.2. Implants

Many of the drugs used to treat diseases of the posterior require repeated administration on a monthly or bimonthly basis, necessitating alternatives to the bolus IVI injection [93]. To further minimize complications and circumvent high clearance rates and the low bioavailability of common drugs, intravitreal implants have been sought after as an alternative [93,94]. These implants can be either biodegradable or semipermanent and are typically made up of a polymeric housing. Compound systems (nano- or microparticles or liposomes contained within a polymeric housing) are also frequently used [95]. In a study comparing the efficacy of a periocular triamcinolone acetonide injection, an intravitreal triamcinolone acetonide injection, and an intravitreal dexamethasone implant to deliver corticosteroids to treat uveitic macular edema, Thorne et al. concluded that the IVI and the intravitreal implant were superior to the posterior injection with a small increase in the risk of IOP elevation [96]. In a comprehensive, retroactive study of 6015 dexamethasone-containing intravitreal implants over an average of 18 months, cataract progression and IOP rise were identified as the most common complications; however, intravitreal implants were considered generally safe with manageable risks [97].

Currently, intravitreal implants are used as an effective treatment for bacterial and viral infections of the vitreous and retina. Since the vitreous is a mostly acellular, heavily hydrated material, it serves as a very effective medium for drug delivery to adjacent parts of the eye. Drugs that are introduced to the vitreous also have less access to the systemic circulation, reducing the risk of nonocular side effects that can arise with treatments such as corticosteroids [98]. A wide range of drug products have seen use in both resorbable and semipermanent implants. Take, for example, the antiviral medication ganciclovir and its accompanying delivery device Vitrasert. Vitrasert is a product produced by Bausch + Lomb and was approved by the FDA in 1996 as a treatment for cytomegalovirus retinitis. Cytomegalovirus retinitis is commonly seen as a secondary infection brought on by the weakened immune system in AIDS patients; approximately 25–42% of patients diagnosed with AIDS will experience the infection [99]. Vitrasert is a polymer drug delivery system that can deliver ganciclovir at a steady rate for up to 5–8 months [99]. The device is composed of two polymers: an outer layer of drug-permeable polyvinyl alcohol (PVA), and an inner layer of impermeable ethylene vinyl acetate (EVA) [98,99,100]. The EVA layer partially encapsulates the inner payload of ganciclovir, effectively reducing the surface area through which the drug can diffuse into the outer polymer. The PVA serves to limit the rate of diffusion between the surrounding vitreous and the device as a whole. This limited rate of diffusion is a key factor in the stability of the device’s release kinetics. The device can deliver a steady and reliable ganciclovir dose of 1 mcg/h without a large initial burst of the drug that can be problematic for the patient [100]. This particular implant is nonresorbable, so it must be removed from the patient’s eye and replaced if the infection persists longer than the product’s dose. Vitrasert is by no means a novel device in its market. There are several other devices operating in the same space, many of which are semipermanent polymer-based designs. While biodegradable alternatives do exist in the market, they are still less prevalent in clinical use and require more research to bring stable, consistent products.

Biodegradable sustained-release intravitreal implants are a particular focus of much research, as they offer the ability to deliver a steady supply of a drug to the vitreous or adjacent structures over an extended period but do not require a removal surgery. Liu et al. reported a composite poly(lactic-co-glycolic acid) (PLGA)-based microsphere loaded into a polyethylene glycol–poly(L-lactide) diacrylate (PEG-PLLA-DA) and N-isopropylacrylamide (NiPAAM) hydrogel.The microsphere-hydrogel composite system loaded with aflibercept was well tolerated, biocompatible, had biodegradable potential, and could treat CNV lesions for 6 months following intravitreal implantation in a rodent model. This device performed comparably to, if not better than, a bimonthly IVI injection and was advantageous in that it required only one treatment [101]. Varela-Fernández et al. described a poly-Ꜫ-caprolactone (PCL) intravitreal implant loaded with idebenone for the treatment of Leber’s hereditary optic neuropathy. This PCL delivery system was well tolerated, biocompatible, degradable, and able to release idebenone for over a year [102]. Systems such as these show much potential to overcome many of the shortcomings of traditional intravitreal drug delivery while providing many additional advantages, including targeted and sustained drug delivery, long-term sustained vitreous drug concentration, and a reduction in treatment frequency.

## 4. The Subretinal Space

### 4.1. Anatomy and Key Cells

There are many causes of blindness; however, some of the most common are those conditions that affect the retina. The retina contains the photoreceptive cells of the eye and can be degraded or damaged by numerous conditions or events. Whether the cause for vision loss is genetic or the result of another conditionsuch as diabetes, one of the golden spots for treatment is the subretinal space [103]. The subretinal space is located next to the photoreceptors that these conditions degrade, making it ideal for quick drug delivery [103].

The subretinal space is located between the retinal pigment epithelium (RPE) and the photoreceptive cells [103]. The majority of the retina is a delicate matrix of photoreceptive cells and their support network which are responsible for human vision. These cells are separated from the cornea by a layer of pigment epithelium. The RPE has tight junctions, effectively insulating the inside of the retina from systemic circulation; the contents of the retina can then be controlled by transcellular transport [104]. This barrier works in tandem with the retinal vascular endothelium to turn the subretinal space of the eye into an immune-privileged space. Due to this, the RPE can exert some control over the immune system by secreting immune-modulatory factors such as interleukin-8 (IL-8), complement factor H (CFH), and monocyte chemotactic protein-1 (MCP1) to activate and deactivate it in response to the disease state of the eye [105,106]. In addition to its immune secretions, the RPE has MHC receptors and toll-like receptors allowing it to respond to signals from the immune system [104]. Besides immune interactions, the RPE routinely secretes growth factors and signaling molecules into the subretinal space and the choroid which are important for ensuring the stability of the photoreceptors and the choroid [104].

The other side of the subretinal space is the photoreceptive cells which are responsible for light detection and are the first layer of the neuroretina [107]. These cells are not actually attached to the RPE; instead, they rest near it, leaving a spot for a subretinal space [108]. This space is a consequence of how the neuroretina forms during embryonic development [108]. The photoreceptors are connected to interneurons, a set of cells that process the raw signal; these connect to ganglion cells that carry visual signals to the brain. The final part of the neuroretina is the glial cells, consisting of Müller cells, astrocytes, microglia, and oligodendrocytes [108]. Müller cells are the most prevalent glial cells in the eye and are found throughout the neuroretina. Their proximal and distal extensions form the inner limiting membrane and the outer limiting membrane, respectively. They are important to maintaining the internal environment of the retina [108]. Astrocytes come from stem cells in the optic nerve and are found in the superficial layers of the neuroretina. Microglia enter the retina from circulation; they are phagocytic cells that are part of the immune system and can migrate throughout the retina [108]. Finally, oligodendrocytes form the myelin sheath of neurons.

The retinal vascular endothelium makes up the other side of the blood–retina barrier via the tight junctions of the retinal blood vessels that prevent fluid leakage [105]. These vessels are responsible for supplying nutrients to the inner eye, where the ganglion and bipolar cells are located [105]. The blood–retina barrier grants the eye immune privilege which severely isolates the subretinal space. However, this isolation can come at a price when immune privilege is compromised. When trauma, infection, or degradation cause antigens to leave the retina, the immune system will not recognize them and autoreactive T cells can be activated [105]. It is also important to mention that immune-privileged does not mean that the subretinal space is completely separated from the immune system. In reality, it mostly protects the retinal tissue from the immune system when it is healthy [105]. During infection, systemic signals and chemokines are released, and activated T cells can enter the subretinal space as easily as any other tissue [109]. Once T cells have entered the retina, they start to accumulate and attract macrophages [105]. The macrophages cause inflammation, which can cause damage to the retina [105].

### 4.2. Interface

#### 4.2.1. Subretinal Injections

There are many approaches currently under investigation for how to deliver drugs and other treatments into the subretinal space. The isolated nature of the retina means that its natural defenses must be breached for any therapeutics to gain entry. Currently, the approaches for administering subretinal injections fall into three categories: (1) a transcorneal route through the pupil and passing the lens, vitreous, and retina [110,111]; (2) a transscleral route entering the pars plana or limbus areas and crossing through the vitreous to enter through the subretina through the opposite side of retina [112,113,114]; and (3) a transscleral route through the choroid and Bruch’s membrane that bypasses the retina [103,115,116,117]. These routes are effective and appropriate for the delivery of viruses, viral particles, liposomes, plasmids, drugs, and formulations and can be used as collection points to measure the contents of the subretinal space [103,118]. However, they have the drawback of causing retinal injury and permanent detachment after several uses [107]. Current research efforts explore the potential of drug delivery systems that would release the drug over a period of time; however, these are all still in their clinical study phase [107]. There are also some concerns over the feasibility of mass-producing these delivery systems due to their fragility and complexity [107].

The effects of subretinal injections on eye tissue are not fully understood. It is known that injections into the subretinal space will result in the formation of a bleb, a temporary detachment of the photoreceptors from the RPE. This separation is necessary for drugs to reach the cells within the retina; however, it also damages the outer retina [119]. Studies have demonstrated that the force from the temporary retinal detachment by injections can alter the photoreceptive and RPE cells. During the detachment, photoreceptors are swollen and fragmented while the RPE cells are damaged, ultimately negatively affecting the ability of the subretina to reattach [120]. There is currently ongoing research in animals to limit the trauma-caused retinal bleb formation; however, there are no standard procedures established to date [120].

Despite their side effects, subretinal injections are used to treat numerous conditions and are seen as a potential method of treatment for many more. Clinically, subretinal injections are used to treat retinal degenerative diseases such as age-related macular degeneration, retinitis pigmentosa, Leber’s congenital amaurosis, and Stargardt disease [103]. There is currently ongoing research for the treatments of these diseases with the use of subretinal injections of viral vector delivery for gene therapy and stem cell delivery for cell therapy [103].

#### 4.2.2. Subretinal Transplants

Subretinal transplants have been performed using RPE and photoreceptive cells, as well as some stem cells [105]. Subretinal transplants are one of the potential ways to treat damaged or degrading retinas; however, they have had limited success [105]. The immune privilege of the retina does not extend to the grafted tissue, so the patient will require the use of immunosuppressants which tend to target the adaptive immune system [105,121]. Despite this, many grafts still have issues with cell survival, as neutrophils and macrophages target and engulf the cells as part of an innate immune response [121]. It is notable that recent phase 1 and 2 trials of subretinal transplants in humans have been more promising. Trials evaluating the success of subretinal injection of pluripotent stem cells showed that the cells were tolerated by the body and a portion of the patients experienced visual improvement for the duration of the trial [122]. Other research has shown that with proper immune suppression photoreceptors could be successfully transplanted and integrated into the subretina [123]. While these results are optimistic, there is still much research to be done in this area.

#### 4.2.3. Retina Prostheses

A retinal prosthesis, a type of bionic eye, is an implantable electronic device designed to stimulate the sensation of vision in the eyes of individuals with significant retinal diseases and is relatively new to the market in both the United States and Europe. This is in part due to new nanofabrication techniques that have allowed for the production of smaller and less invasive devices [124]. While many devices are fixed onto the surface of the retina, some are placed into the subretinal space, which removes the need for device fixation. The perceived advantage of subretinal implants is that the device is implanted where the degenerated photoreceptors are, allowing the system to take advantage of the natural retinal structures and to have greater similarity to physiological systems [124]. However, the photoreceptor systems that these devices are trying to take advantage of are often damaged by disease or through device implantation [124]. It is also notable that devices are challenging for surgeons to implant due to their location and the underlying degeneration that results in unwanted adhesion to the retina and retinal pigment epithelium [124].

There are several subretinal implants currently on the market or in testing. The earliest was the Boston Retinal Implant Project, which used single electrode stimulation to treat retinitis pigmentosa and orbital cancer. While this device does not produce functionally useful vision, its developer does have a more advanced device in clinical trials [124]. This device notably needed an external power source to function. Another device is the Artificial Silicon Retina which is supposed to stimulate the retina in response to ambient light by converting it into an electrical signal using the ambient light as its power source [124]. This device is a silicon retina array with 5000 micro-photodiodes and iridium-tipped microelectrodes and is implanted into the superior retina. In the trial study of this implant, four of six patients could detect phosphenes, which are light spots in the visual field of the implant. It is also notable that some patients experience visual enhancement outside of the field of implant, suggesting that this device can influence nerve growth in the retina [124]. This device was ultimately concluded to not produce sufficient photocurrent to stimulate neurons from ambient light alone. [124].

The Alpha IMS and AMS are the only subretinal devices approved for sale in Europe. Like the Artificial Silicon Retina, the Alpha IMS uses a photovoltaic array consisting of a microchip with 1500 photodiode-amplified electrodes [124]. However, this device also uses an external power source to amplify the signal it produces from light, giving it an advantage over the Artificial Silicon Retina [124]. This external power reaches the implant by a silicon cable linked to a fixation pad in the orbit. Some users of this device were able to sense motion, while approximately 20% could see letters or objects and about 30% saw no visual improvement; the rest were able to perceive light and had improved ability to localize it. It is notable that the object recognition only improved for about 3 months but eventually fell significantly [124]. The Alpha AMS is a 1600 photodiode system that has improved longevity over the original [124].

Finally, the Photovoltaic Retinal Implant (PRIMA) Bionic Vision System relies on pixels that receive near-IR light pulsed from a pair of glasses which is used to stimulate an electrode [124]. This electrode stimulates another electrode connected to iridium oxide coated photodiodes, which then stimulate the adjacent neural tissue [124]. This allows for improved spatial resolution and scalability without requiring additional wires. For PRIMA, animal testing results have been promising, and human clinical trials are underway [124].

### 4.3. Current Research

#### 4.3.1. Gene Therapy

The use of viral vectors to edit the genome is being investigated as one of the ways of treating inherited retinal disease. These treatments would be placed into subretina as it would allow the vectors to target the photoreceptor or RPE cells while limiting the immune response and dosage needed [103]. Studies already completed in animals suggest that the adeno-associated virus (AAV) is a feasible method for longer-term gene expression in the retina; continued work on this vector will allow it to be applied to more diseases and improve efficiency [125]. It is the most common method for delivering genetic material to the retina [103]. It has been used to successfully target both RPE and photoreceptor cells for the treatment of various degenerative conditions in animal models [103]. Other vectors, such as helper-dependent adenoviral vectors, have been used to improve AAV’s abilities [103]. Lentiviral vectors have also been used for gene therapy of the retina [103]. While most of the work thus far has focused on genetic and degenerative conditions, some work has also been done on treating autoimmune uveoretinitis in animals [103]. For humans, AAV-based treatments are in clinical trials; these tests have shown that AAV treatments are not systemically toxic and there are no serious adverse events associated with their use, as well as showing promising improvements in patients’ vision [103]. It is notable that these treatments are administered by subretinal injection [103].

#### 4.3.2. Cell Therapy

Cell therapy is the placing of cells into the subretina, generally by subretinal injection and recently via a microcatheter, to treat retinal degenerative diseases [103,126]. These systems typically involve the injection of stem cells intended to integrate into the retinal layers and help restore function or support cell regeneration; however, sometimes other photoreceptive or RPE cells have also been used [103,123] While animal studies have suggested that this technique is safe and nontoxic, there are concerns over the high risk of complications. Currently, some phase 1 and 2 studies are ongoing [103]. Gandhi et al. at the Mayo Clinic recently demonstrated the safety and efficacy of degradable fibrin hydrogels for subretinal implantation to facilitate the precise and uninterrupted implantation of an RPE monolayer [127]. These promising hydrogels effectively degraded from the space in 8 weeks following delivery and represent the first fully degradable scaffold developed to treat macular degeneration and degenerative diseases of the retina [127].

#### 4.3.3. Novel Delivery Methods

Due to the segregated nature of the subretina, there is much research interest in exploring how to successfully deliver drugs through minimally invasive means. While there is a large variety of methods for drug delivery under investigation, this review will limit itself to the more common ones. One of the routes considered is the use of nanoparticles to assist in drug delivery. Nanoparticles could be used to protect the drug and transport it through the blood–retina barrier or to allow it to have sustained release. Cerium oxide nanoparticles have been used to scavenge reactive oxygen species in the eyes of mice, thus preventing oxidative stress and serving as a proof of concept for their ability to slow disease progression [128]. Nanoparticles can also be used to encapsulate DNA or RNA and aid in its uptake into retinal cells without the use of a viral vector [129]. This would allow for a different means of gene therapy for the cells in the eye [129]. In terms of increasing drug dosage and extending drug delivery time, research has shown that nanoparticle encapsulation can be used to deliver hydrophobic compounds to RPE cells over an extended period [85].

Liposomes are another potential way to deliver drugs to the subretina, as their hydrophobicity would allow them to cross the blood–retina barrier by means of diffusion. PEG molecules have been used to encapsulate drugs for delivery to the brain across the blood–brain barrier, a similar anatomical barrier [130]. Given the similarities between the blood–brain barrier and the blood–retina barrier, there is a good chance such a system would also be effective for drug delivery to the subretinal space [130].

Injectable hydrogels are a potential means of long-term drug delivery in the subretinal space. Hyaluronic acid hydrogels have previously been used to transplant retinal progenitor cells into the subretinal space [131]. The transplanted cells were distributed evenly within the subretinal space after three weeks and had shown characteristics indicative of maturation into photoreceptors [131]. Hydrogels could serve as a method for the long-term release of drugs and biologics into the subretinal space [132]. Their use would negate the need for repeated subretinal injections; however, it would still require the formation of a bleb and introduce trauma to the eye.

Another means of allowing drugs to enter the subretinal space would be modulating the blood–retina barrier. This would be accomplished by creating temporary openings in the membrane. After this, drugs can enter from the bloodstream, thus negating the need for repeated injections. This has previously been done by siRNA-mediated knockdown of a protein related to the function of the tight junctions between the cells of the barrier [133]. Targets for this are claudin-5, which is one of the three proteins that make up tight junctions [133], and occludin, another protein component of tight junctions that was targeted in the blood–brain barrier [134]. While this means of drug delivery would not be appropriate for chronic conditions, it could be used for one-time delivery, like those needed for gene therapy [107].

## 5. The Subconjunctival Space

### 5.1. Anatomy and Key Cells

The subconjunctival space is the hydrophilic, fluid-filled space between the conjunctiva and the sclera. Additionally, the subconjunctival space has access to all the blood vessels found in the conjunctiva, which can help to further distribute substances throughout the whole eye. The subconjunctival space is located superior to the cornea and optimally located to distribute drugs to several different parts of the eye through minimally invasive means while limiting the development of scar tissue [22,135].

### 5.2. Interface

When considering periocular drug delivery via the subconjunctival space, it is critical that the agents and delivery systems do not react with the subconjunctival fluid, causing irreversible damage to the eye. This subconjunctival fluid-like gel is, like the vitreous humor, predominantly composed of water with a small percentage of hyaluronic acid, glucose, ions, and collagen [136,137]. The subconjunctival space is pressure-sensitive due to its flexibility and resistance to fluid dissipation, making it essential that pressure be monitored when administering drugs or drug delivery systems to this region [138]. Additionally, conjunctival and choroidal circulation potentially reduce the ocular bioavailability of drugs permeating from this region [22]. The retinal pigment epithelium, chemosis, and risk of subconjunctival hemorrhage pose additional challenges to this delivery route [22].

#### 5.2.1. Drug Delivery

The subconjunctival route is appropriate for drug delivery to both the anterior and posterior segments of the eye [22]. Drugs administered through the subconjunctival space circumvent the cornea, conjunctiva, and the conjunctival–epithelial barrier, passing directly through the sclera into the posterior segment [20,22,139]. Studies suggest that when drugs are administered into the subconjunctival space in a free, unencapsulated form, they may be delivered at a relatively low concentration due to the ease of travel to adjacent locations. Weijtens et al. (1999) demonstrated that a subconjunctival injection of only 2.5 mg of dexamethasone disodium phosphate in humans resulted in a mean vitreous dexamethasone peak concentration that was 3 and 12 times higher than that after 5 mg and 7.5 mg doses administered by peribulbar and oral administration routes, respectively [139]. However, researchers also suggest that following subconjunctival administration, colloidal dosage forms (up to 20 nm in size) and released drug molecules are rapidly cleared by the conjunctival, choroidal, and lymphatic circulations, thereby limiting ocular bioavailability [22,139,140,141]. Additionally, free-form drug injections must be rate-limiting to avoid overdose [22]. Currently, the main types of drugs administrated through the subconjunctival space are carboplatin, topotecan, insulin, and other drugs with similar chemistries [22].

##### Drug Delivery Systems

Along with predominantly free drug injection, there have been some preliminary trials involving sustained-release drug delivery systems. These systems are generally microparticles, nanoparticles, and collagen matrices which have been found to be too large to fit between pores into the retina and do not easily break down [22]. The motivation behind developing these systems is to house and deliver drugs that would have long-lasting effects; therefore, each of the systems releases at a different rate and appropriately administers the drug in question. Unfortunately, due to the slow rate of degradation and poor clearance of the housing material after drug delivery is complete, there have been several logistical challenges with implementing these systems. *Liu* et al. found success with biodegradable poly(lactide-co-ɛ-caprolactone) microfilms loaded with prednisolone and implanted in the subconjunctival space of a rat model, showing these systems to deliver the drug for 3 months at a rate of 0.002 mg/day [142]; several similar systems are under development.

A promising approach to drug delivery systems designed for the subconjunctival space is through liposomes [143,144], polymeric thermoresponsive hydrogels [94,145,146], and polymeric controlled-release systems [34,147] that would release drugs and subsequently degrade. These drug delivery systems limit the amount of drug lost compared to a free drug injection, but also break down into small enough particles that they can travel through the small pores. They can also last longer since the capsule units will degrade at varying rates. Drug delivery systems for the subconjunctival space are taken advantage of to improve the patient experience.

##### Liposomes

Liposome drug delivery treatments for the subconjunctival space are being evaluated for how quickly the drugs can be transmitted into the subconjunctival space and subsequently diffuse into other areas of the eye while maintaining a relatively large concentration. In one study evaluating tobramycin liposomes, Assil et al. used negatively charged liposomes to deliver tobramycin to infected rabbit eyes [143]. This study found that liposomes allowed for higher, more rapid peaks of the drug compared to topical treatments. They also found that they were able to sustain drug delivery for 24 h in the cornea after the liposomes were administered to the subconjunctival space. After 24 h, the drug concentration dropped dramatically, suggesting that liposome treatments would require frequent administrations for long-term illnesses, which could have growing complications [143].

Another study used negatively charged liposomes to deliver gentamicin to infected rabbit eyes [144]. This study revealed that not all parts of the eye received the drug, and in those parts that did, the drug was unequally distributed, and gentamycin levels were higher in the sclera and cornea than when gentamycin was injected in its free form. This study also confirmed the inability of negatively charged liposomes to migrate throughout the ocular structures, partially also due to their large size. This result may differ with a positive charge [144].

From both of these experiments, it is clear that liposomes cause a rapid peak in drug concentration when first administrated, which could be useful when combating an initial infection that would require additional follow-up injections. Moreover, liposome subconjunctival drug delivery was effective at delivering drugs more posteriorly as compared to topical treatments. For targeted drug delivery, liposomes are a promising method to provide a rapid and high concentration in specifically targeted ocular regions.

##### Hydrogels

Environmentally responsive hydrogels offer another promising option for sustained-release drug delivery into the subconjunctival space owing to their ability to be injected into the space and offer slow and controlled drug release without a risk of migration. Thermoresponsive hydrogels can be injected into the space through a small gauge needle at room temperature and proceed to collapse into a more solid form upon reaching body temperature, promoting the release of encapsulated drug [145,148]. Additionally, these hydrogels can be made biodegradable, eliminating the need for a removal surgery [149]. In a 2008 study, Kang Derwent (Kang-Meiler) and Mieler demonstrated the ability of thermoresponsive PEG and poly(N-isopropylacrylamide) (NiPAAM)-based hydrogels to be manipulated to control their drug release rate, confirming the absence of an immune response. Kang-Mieler also established that while the hydrogels themselves did not migrate, the encapsulated drug was able to travel to the posterior region of the eye [146]. Dosmar et al. demonstrated the use of these hydrogels loaded with vancomycin and injected into the subconjunctival space to prevent acute endophthalmitis in the vitreous following ocular surgery in male rodents [145]. These hydrogels released detectable levels of vancomycin at a steady rate for nearly three weeks.

##### Polymeric Controlled-Release Systems

Polymeric controlled-release systems for the subconjunctival space are less common due to their larger size and the fact that they do not typically biodegradable. While more frequently used on the ocular surface, several labs have developed polymeric systems for the subconjunctival space. Cui et al. used 5-fluorouracil-loaded poly(lactic acid) discs implanted into the subconjunctival space after glaucoma filtration surgery in rabbit eyes. The discs sustained drugs throughout the critical period for 2 weeks until 1 month where they failed to administer additional drug [150]. Animals experienced some subconjunctival hemorrhage, which was attributed to the disc implantation. Different polymer mixtures resulted in toxic effects that cause conjunctival hyperemia and corneal edema [150]. Zignani et al. experimented with two different anti-inflammatory drugs (dexamethasone sodium phosphate and 5-fluorouracil (5-FU)) to see which one would reduce the harmful effects of hydrophobic poly(ortho ester) on the subconjunctival space [151]. The study revealed the promising effects of dexamethasone to abate the toxic effects of a typically reactive polymer, making it possible for use as a long-term drug delivery system [151].

## 6. Material Interactions

Interaction between a drug carrier and the ocular environment caused by the carried drug is an important topic for consideration when discussing sustained release drug delivery systems. Due to the vast amount of materials available for drug carriers, summarizing the general interactions between drugs and all carriers is infeasible. Instead, this article will categorize the materials and discuss some specific interactions within each category with a special emphasis on hydrogels.

Chitosan and alginate are the most common natural materials used in drug delivery. Chitosan is an extract from chitin found in the cell walls of fungi and shells of arthropods, which is then treated with a deacetylation process [152]. Alginates are produced by Pseudomonas [153] and are obtained from dehydrating algae by brine drying [154]. Synthetic materials, on the other hand, are based on various monomers and polymers. Renowned examples include polyethylene glycol (PEG), PLGA, and NiPAAM. This review will analyze some material interactions between the materials and the ocular space.

### 6.1. Chitosan

Chitosan is only soluble in an acidic environment [155], meaning that for most infections where acidosis occurs in the ocular space (pH 7.11) [156], a chitosan hydrogel can function normally. Therefore, chitosan as a material should not be used for controlled release in neutralizing alkaline environment, as the material will react with the acidic reagent inside, causing bulk erosion. Due to a tight pH limit on the material, modifications to chitosan hydrogels are a new topic with this material. PEG and glycerol are some robust candidates, enhancing the material with some additional antibacterial effects against E. coli and S. aureus [157], while Chenite et al. discovered that chitosan–β-glycerol phosphate could also dissolve in pH around 7.2 [158]. This indicates the possibility of using such material for drug delivery under normal ocular pH.

### 6.2. Alginates

Different from chitosan, alginates are anionic. The molecular structure of alginate ensures that controlled drug release is available in neutral and basic solutions. Jao et al. experimented with calcium alginate hydrogels and confirmed that in simulated gastric fluids, only 20% of the total loaded drug was released. Swelling was also observed during this process [159] suggesting that acidic environments are not optimal for alginates to function.

### 6.3. PEG

PEG is soluble in both water and organic solutions. By linking to other hydrophobic molecules and conjugating with them, PEG reduces the immunological response and increases the solubility of the target molecule [154,160]. However, Środa et al. found that PEG increases protein adsorption, suggesting some inhibitory effect on PEG-bound proteins [161]. Armstrong et al. also reported that patients developed antibodies against PEG after being injected with PEGylated asparaginase [162]. Although none of the adversity reports mentioned above involved the ocular space, the use of PEG should be exercised with care and specific research.

### 6.4. PLGA

Poly(lactic-co-glycolic acid) (PLGA) is a biomaterial that is copolymerized from poly(lactic acid) and poly(glycolic acid). It does not require removal after all drug has been released [163]. Its greatest potential lies in fabricating multidrug delivery devices, where multiple pulses of burst release are desired. Cleland et al. used different PLGA concentrations on PLA polymers to mimic the effect of a single-shot subunit for an HIV-1 vaccine [164]. However, it should also be noted that PLGA itself without treatment and enhancement from other materials is not an optimal carrier, especially for antigens. As a bulk-eroding system, the material’s debris from the erosion might interfere with antibody active sites [165].

### 6.5. NiPAAM

NiPAAM usually forms a hydrogel with a copolymer. Chitosan/NiPAAM, poly(N-isopropylacrylamide-co-dimethyl-γ-butyrolactone acrylate-co-acrylic acid) (poly(NDBA)), and poly(N-isopropylacrylamide-co-sodium acrylate) (PNiPAAm-co-PNaAc) are some common NiPAAM based copolymers [166]. Raju et al. used a bilayer hydrogel, both layers consisting of NiPAAM-based copolymers, and reported good encapsulation efficiency with L-DOPA [167]. Dosmar et al. reported similarly good encapsulation (>84%) with vancomycin and demonstrated the biocompatibility of PEG-NiPAAM hydrogels [145]. In broad terms, NiPAAM provides temperature-dependent control to a hydrogel due to a lower critical solution temperature occurring at a value similar to body temperature. This means that as it is implanted into the body and contacts the bodily fluids, the NiPAAM is miscible, allowing drug delivery [168].

## 7. Anti-VEGF Drugs

In addition to the medical implants already discussed, we also explored three pharmaceutical anti-VEGF treatments and their pharmacokinetics within the body: aflibercept (Eylea), ranibizumab (Lucentis), and bevacizumab (Avastin). Anti-VEGF treatments target the vascular endothelial growth factor (VEGF) and its receptors which take part in both normal and pathological angiogenesis. The activation of the VEGF receptor pathway leads to the promotion of endothelial growth and migration, potentially leading to ocular pathologies such as age-related macular degeneration, proliferative diabetic retinopathy, central retinal vein occlusion, and choroidal neovascularization [169,170]. Anti-VEGF therapies have shown significant promise in their ability to inhibit VEGF while remaining nontoxic to both the retina and optical nerves. Therefore, comparison of the different Anti-VEGF drug categories is an important part of understanding different biomedical implantation strategies [169,171,172].

### 7.1. Eylea

Eylea is a soluble decoy receptor consisting of the second Ig domain of human VEGFR1 and the third Ig domain of VEGFR2, as well as the Fc region of VEGF-A [173,174]. As an iso-osmotic drug, it is injected directly into the intravitreal space for the treatment of wet age-related macular degeneration [172,175]. In contrast to both Lucentis and Avastin, Eylea binds not only multiple isoforms of VEGF-A, but also to the ligands VEGF-B and PlGF [173]. It has been shown that Eylea has a higher binding affinity for VEGF-A (~490 pM) than both Lucentis (46 pM) and Avastin (58 pM), which is consistent with an increased potency in inhibition of VEGFR1 and VEGFR2 via VEGF-A [172]. Eylea has been shown to have the greatest impact on free-VEGF levels in the body when compared with both Lucentis and Avastin [176].

### 7.2. Avastin

Avastin is a recombinant humanized monoclonal IgG1 antibody that binds and inhibits VEGF-A. While its original purpose was the treatment of metastatic colorectal cancer, it has shown promise in helping VEGF-mediated diseases when injected intravitreally [169,177]. Avastin has been shown to have a half-life of around 4.32 days in the rabbit model with a maximum serum concentration being reached in the vitreous after 8 days. Studies have shown it to have a lack of retinal toxicity even when injected in higher concentrations, though some inflammatory cells were found at a 5 mg dosing in rabbits [169,170].

### 7.3. Lucentis

Lucentis is a recombinant humanized monoclonal IgG1 antibody fragment derived from Avastin and developed specifically for the purpose of treating intravitreal eye diseases [171,178,179]. It has a similar but slightly lower binding affinity for VEGF-A than Avastin [172] and has shown great promise in clinical trials in reducing vision loss, and even assisting in some vision improvements for patients [178]. Lucentis has been shown to have a half-life of 2.88 days in the vitreous of a rabbit model, with a maximum concentration being reached after 3 days. Due to its relatively smaller molecular size as compared to Avastin, it is likely that Lucentis can more easily penetrate the retina and be cleared from systemic circulation, though there are differences in tissue distribution for both drugs [171].

Papadopoulos et al. tabulated the kinetic binding parameters for each of these drugs when binding the VEGF ligands [172]. While the results indicate that Eylea is the optimal choice for treatment of macular degeneration and other pathologies of the eye, there are a few other drugs of note that have not been discussed at length in this section. Macugen is another anti-VEGF treatment that has been associated with positive outcomes in the treatment of diabetic macular edema in phase 2 studies [180,181]. Visudyne is a medication used in photodynamic therapy that can help to reduce vision loss due to macular degeneration [182], and Beovu is a VEGF inhibitor that functions in many of the same ways as Eylea, Avastin, and Lucentis [183].

## 8. Conclusions and Future Directions

In this review, we have discussed four primary routes for ocular drug delivery, namely topical, intravitreal, subretinal, and subconjunctival, in the context of the ocular anatomy, biological interface, primary barriers, and the state of current research. In addition, we have touched on their respective advantages and limitations and where their use is most appropriate. To enhance the usefulness of this information, we have also included sections on the interactions of materials and pharmaceuticals with the ocular space. The information found in this review can be found summarized in Table 1.

As discussed, ocular drug delivery remains at the forefront of drug delivery research. With the barriers of topical delivery continuing to pose technical challenges and the limitations of repeated dosing, sustained-release technologies, and implants have emerged as attractive alternatives to single-dose administration of treatments. Noteworthy are the devices that allow for the complete release of a pharmacologic over a period of weeks to months and then degrade completely without the need for a removal procedure. These devices circumvent the challenges associated with repeated injections as they are overall less invasive, more convenient, and could minimize infection. It is expected that the market will see a continued rise in the use of polymeric-based sustained-release drug delivery devices and refillable ports such as these as they pass phase 1 and phase 2 clinical trials. It is also expected that retinal prosthetics will emerge prominently on the market as the technology progresses. In general, approaches to ocular drug delivery will seek to optimize drug retention and performance while minimizing the invasiveness of the procedure. Many of the devices evaluated in this review are in their early stages of study and clinical trials. However, we expect that within the next decade, the market will see a significant increase in the availability of novel drug delivery devices as increasing numbers gain Federal Food and Drug Administration (FDA) approval in the United States and Conformitè Europëenne (CE) approval in Europe.

## Figures and Tables

**Table 1:** Summary of the primary delivery modalities including the type of delivery, advantages, disadvantages, and noteworthy materials used.

Location	Type of Delivery	Purpose	Advantages	Limitations	Noteworthy Materials
*Topical*	Topical liquids and solutions	Antibiotics, anti-inflammatory and, antifungal drugs	Noninvasive	Significant drug loss before internal penetration, treatment must be isotonic with tears	Viscosity enhancers, permeation enhancers, and cyclodextrins
	Emulsions and microemulsions	Glaucoma, uveitis, keratitis, and ocular bacterial and fungal infections	Improved drug stability, permeation, residence time, and bioavailability compared topical liquids; effective at delivering poorly water-soluble drugs, transparent	Large quantity of surfactant required to form stable microemulsions, which can cause ocular toxicity	Tween 60 and Tween 80
	Suspensions and nanosuspensions	Antibiotics, anti-inflammatory and, antifungal drugs, increasing bioavailabilty of hydrophobic drugs	Appropriate for delivery of insoluble pharmaceuticals; have been shown to improve bioavailability of hydrophobic drugs	Physical stability and the potential for drug sedimentation	Viscosity enhancers
	Ointments	Vancomycin to treat bacterial conjunctivitis; blepharitis, conjunctivitis, and keratitis caused by MRSA and MRSE	Improved bioavailability and sustained release	Limited applications	Semisolid and solid hydrocarbon
	Contact lenses and hydrogels	Increased drug residence times in the tear film and continuous drug delivery	Increased drug penetration; >50% bioavailability in comparison to traditional eye drop	Surface roughness can increase bacterial adhesion; drug loss during storage; limited shelf life; transparency	Silicone and/or polyhydroxyethyl methacrylate (HEMA), poly(lactic-co-glycolic acid) (PLGA)
*Intravitreal*	Injections	Retinal detachment, retinal hemorrhage, antibiotics, steroids, gasses, triamcinolone acetonide, anti-VEGF drugs	Maximize dosing in the vitreous and retina	Endophthalmitis, ocular pain, subconjunctival hemorrhage, and elevated intraocular pressure (IOP); low risk of subretinal hemorrhage, retinal toxicity, and retinal	
	Implants	Conditions of the posterior segment, triamcinolone acetonide, dexamethasone, corticosteroids to treat uveitic macular edema, bacterial and viral infections, CNV, idebenone for the treatment of Leber’s hereditary optic neuropathy	Minimize treatment, minimize complications, circumvent high clearance rates and low bioavailability	Elevated intraocular pressure (IOP), cataract progression	Degradable or semidegradable polymer, PVA, PLGA, NiPAAM, PCL, chitosan, alginates
*Subretinal*	Injections	Appropriate for the delivery of viruses, viral particles, liposomes, plasmids, drugs, and formulations to treat age-related macular degeneration, retinitis pigmentosa, Leber’s congenital amaurosis, and Stargardt disease	Bypass major barriers including the blood–retina barrier	Potential for retinal injury and permanent detachment after several uses, damage to the outer retina due to bleb formation	
	Transplants	Used to treat damaged or degrading retina	Restoration and support of photoreceptor cells	Trigger innate immune response	RPE, photoreceptive cells, some stem cells
	Retinal prosthetics	Vision restoration	Similarity to physiological systems	Challenging to place	
	Gene therapy	Inherited retinal disease	Close access to photoreceptor or RPE cells while limiting the immune response and dosage	Early stages, limited applications	Vectors
	Cell therapy	Retinal degenerative diseases, macular degeneration	Close access to photoreceptor or RPE cells	Early stages, concerns over potential risk and complications	
	Nanoparticles	Conditions of the photoreceptor and RPE cells, drug delivery to the vitreous	Protect the drug, bypass blood–retina barrier, allow sustained release, encapsulate DNA or RNA without the use of a viral vector, deliver hydrophobic compounds	Early stages, shelf life	Cerium oxide nanoparticles
	Liposomes	Conditions of the photoreceptor and RPE cells, drug delivery to the vitreous	Bypass blood–retina barrier, sustained release	Early stages, shelf life	PEG
	Hydrogels	Conditions of the photoreceptor and RPE cells, drug delivery to the vitreous	Bypass blood–retina barrier, sustained release	Need for injectability, bleb formation	Hyaluronic acid
*Subconjunctival*	Liposomes	Antibiotic delivery	Drug retention, sustained release	Potential need for multiple treatments	
	Hydrogels	Antibiotic delivery	Environmentally responsive, injectable, drug retention, sustained release, no migration	Need for degradability	NiPAAM, PEG, PLLA
	Polymeric controlled-release systems	Antibiotic and anti-inflammatory drug delivery	drug retention, sustained release	Need for degradability, hemorrhage, toxic effects that cause conjunctival hyperemia and corneal edema	PLA, poly(ortho ester)

## Data Availability

Not applicable.
